# Regulatory and Educational Initiatives to Prevent Food Choking Injuries in Children: An Overview of the Current Approaches

**DOI:** 10.3389/fpubh.2022.830876

**Published:** 2022-05-19

**Authors:** Giulia Lorenzoni, Alexander Hochdorn, Giulia Beltrame Vriz, Andrea Francavilla, Romina Valentini, Solidea Baldas, Giselle Cuestas, Hugo Rodriguez, Achal Gulati, A. B. Sebastian van As, Dario Gregori

**Affiliations:** ^1^Unit of Biostatistics, Epidemiology and Public Health, Department of Cardiac, Thoracic, Vascular Sciences and Public Health, University of Padova, Padova, Italy; ^2^Department of Social and Work Psychology, University of Brasília, Brasília, Brazil; ^3^Department of Child and Mothers, Santa Chiara Hospital, Trento, Italy; ^4^Dietetics Unit, Department of Medicine, University of Padova, Padova, Italy; ^5^Prochild Onlus, Trieste, Italy; ^6^Hospital de Pediatría Juan P. Garrahan, Buenos Aires, Argentina; ^7^Department of Otorhinolaryngology (E.N.T.), Maulana Azad Medical College, New Delhi, India; ^8^Trauma Unit, Red Cross War Memorial Children's Hospital, University of Cape Town, Cape Town, South Africa

**Keywords:** food, choking injuries, prevention, children, recommendations

## Abstract

Choking injuries are one of the major causes of death among children ages 0–3, and most of these injuries are related to food. This work provides an overview of the current recommendations for food choking prevention and educational targets as a basis for developing a unified common set of knowledge for primary prevention policies development. Guidelines published by professional membership organizations and national governments in the English language were considered. All of these guidelines provide lists of hazardous food items and recommendations for food preparation to minimize choking hazard. Together with recommendations for food preparation, also recommendations aimed at stakeholders (food manufacturers, health care providers, and public authorities) are provided, underlining that this severe public health problem should be further addressed by adopting integrated public health interventions. Our overview stressed the importance of developing educational and primary prevention policies to sensitize adult supervisors and to regulate dangerous food products in the market.

## Introduction

Choking is recognized as one of the major cause of death for infants and toddlers aged 0–3, but the incidence rates are still relevant (1) even well above that age and are currently recognized as a relevant public health challenge (2).

Food plays a major role among objects causing these injuries (2). Furthermore, data from the Susy Safe database (3), perhaps the largest international collection of data on foreign body injuries in children aged 0–14, indicate that adult supervisors lack proper information, a fact also confirmed by a recent survey conducted on parents of children under the age of four (4). Indeed, ~40% of food-related injuries occur in the absence of adult supervision while the child is eating. The remaining 60% occur with adult supervision but with the children having been served improperly prepared or unsuitable food (5).

One of the main reasons promoting choking in the pediatric age could be a lack of parental attention (6). In recent years, several position papers, guidelines, and recommendations have been produced by academics and public health bodies to properly inform and guide adults in the age-appropriate administration of food to infants and toddlers. However, although food preparation is an important component of nutrition science, the subjects of the adequate preparation of food and the proper serving to children to reduce the risk of choking have not yet received sufficient attention (7).

Notwithstanding, empirical surveys seem not to take psychological processes and sociocultural background seriously into consideration. However, studies on other non-communicable diseases in early childhood showed how cognitive functions, especially selective attention, along with emotional-driven factors on the one hand and sociodemographic and educational factors on the other, exercise a deep impact on strictly healthcare-related issues. Among these studies, researches about pediatric overweight and obesity clearly evidenced the influence of such psychological and sociocultural variables (8, 9). Indeed, these studies individuated that appropriate education and information of the general population regarding diseases represents one of the most efficient prevention methods.

Furthermore, other studies showed how sociocultural factors, such as, socioeconomic background and educational levels, are significantly relevant for an adequate understanding and exhaustive comprehension of healthcare-related diseases (10). As emerged from former research (11), the most disadvantaged social classes have less access to higher education, social integration, and appropriate healthcare policies (12). Such conditions might promote negligence and a lack of attention during parental caregiving and surveillance activities regarding younger children.

This paper aims to provide an overview of the current recommendations as a basis for developing a common set of knowledge for developing primary prevention policies of food choking injuries in children.

## Materials and Methods

Guidelines providing recommendations for the prevention of food choking in children (e.g., which food items are the most hazardous, how to prepare them to reduce the risk of choking) were considered. Only guidelines published by professional membership organizations and national governments in English language were included. Recommendations promoted by other organizations (e.g., websites providing informal information to parents and families about potentially dangerous food items) were excluded, as they usually appeared to be reformulations of the official ones. However, some of them, such as Safe Kids Worldwide (13), Nationwide Children's Hospital (14), appeared to be of high quality and promoted high-impact tips for preventing food choking.

To retrieve the existing guidelines, PubMed was queried using the following key terms: food, choking injuries, suffocation, and prevention. No time limits were applied. The same words were employed to extend the search to government websites and other non-indexed search engines. However, because the search of non-indexed search engines was only partially performed, the results from it that are included in the overview are not intended to be exhaustive.

## Results

According to such criteria, 11 recommendations were retrieved that were published between 2000 and 2018 (Table 1).

**Table 1:** Summary of the guidelines retrieved.

**Institutional guideline**	**Nation**	**Year**	**Age for which the recommendations were developed**	**Dangerous food items listed**	**Food preparation**	**Behavioral rules/recommendations besides food preparation**
Ministry of Health (20)	New Zealand	Published in 2008, partially revised in 2012	Under 5 years of age	- Small hard foods (e.g., nuts, large seeds, popcorn husks, raw apples, carrots and celery)	- Do not give candies, bubble gum, or marshmallows to children under the age of 3	- Supervise mealtimes
				- Small round-shaped foods (e.g., grapes, berries, raisins/sultanas, peas, watermelon seeds, candies)	- Remove skins and fibers	- Do not allow children to play and run with food in their mouths
				- Foods with skins or leaves (e.g., sausages, chicken, lettuce, nectarines)	- Cook food until is soft	- Require children to eat at the table
				- Compressible foods (e.g., hot dogs)	- Thinly spread thick pastes	- Never force children to eat
				- Thick pastes (e.g., chocolate spreads, thick peanut butter)	- Cut dangerous food items into small pieces	- Require children to eat in a high-chair and away from distractions
				- Fibrous or stringy foods (e.g., celery, raw pineapple)		- Learn first aid for dealing with choking.
						- Offer food that matches each child's chewing and grinding skills
U.S. Department of Agriculture and U.S. Department of Health and Human Services (Nutrition and Wellness Tips for Young Children: Provider Handbook for the Child and Adult Care Food Program) (21)	US	2012	Under 4 years	- Food smaller than a nickel - Smooth, or slippery foods (e.g., cherries, berries, canned fruit, peanuts and nuts, hard or round candy, jelly beans) - Small, dry, or hard foods (e.g., potato and corn chips, apples or other hard pieces of raw fruit, peanuts, nuts, and seeds) - Sticky or tough foods (e.g., pieces of uncooked or dried fruits or vegetables, fish with bones, chewing gum, sticky candies)	- Cook food until it is tender and then mash it with a fork - Chop foods into small pieces - Always remove bones from fish and meat - Remove seeds	- Kids must stay seated during the meal - Create a quiet environment - Supervise the child while eating - Children should eat slowly
					- Thinly spread peanut butter or seed butter	- Give food for which children are developmentally ready
						- Do not give food in the car
American Academy of Pediatrics (15)	US	2010	Under 4 years, even if it has been noted that the problem is relevant until 14 years (with 10,000 emergency department visits)	- Cylindrical, airway sized, and compressible foods (e.g., hot dogs), hard candy, peanuts/nuts, seeds, whole grapes, raw carrots, apples, popcorn, chunks of peanut butter, marshmallows, chewing gum, and sausages.		Activities recommended to the FDA for food choking injury prevention - Surveillance of the phenomenon - Sensitizing campaign about the problem of choking injuries - Warning labels for dangerous food products - Recalls of dangerous food products from the market Additionally: - Health professionals should provide choking prevention counseling to families
						- Food manufacturers should redesign hazardous products
Canadian Pediatric Society (16)	Canada	2012	Under 4 years	Small, round, cylindrical shaped foods (e.g., hot dogs, peanuts, seeds, hard candies)	- Food that should be avoided under the age of 4: hard candies, cough drops, gum, gummy candies and chewable vitamins, peanuts, sunflower seeds, fish with bones, snacks on toothpicks or skewers - Foods that could be given to children if adequately prepared: grapes, hot dogs and carrots (chop lengthwise); raw carrots, apples (cut or finely grate)	- Surveillance of the phenomenon - Develop legislations and standards (and monitoring the compliance) - Improving product design
						- Counseling to families and choking prevention campaigns
U.S. Department of Agriculture (Building Blocks for Fun and Healthy Meals: A Meal Planner for the CACFP) (22)	US	2000	Under 4 years	- Small hard foods (e.g., nuts, seeds, popcorn, pieces of raw vegetables)	- Cook food until tender	- Sit quietly while eating
				- Tough or sticky foods (e.g., peanut/seed butters, marshmallows, bubble gum)	- Cut round foods lengthwise	- Serve small portions
				- Firm, smooth or slippery foods (e.g., hot dogs, hard candies, cherries)	- Chop food into small pieces	- Encourage children to eat slowly and chew well
					- Remove bones, seeds and pits	
					- Thinly spread peanut butter	
U.S. Department of Agriculture (Feeding Infants: A Guide for Use in the Child Nutrition Programs) (23)	US	2001	The guide was aimed at infants	- Small, dry or hard foods (e.g., popcorn, peanuts, pieces of raw fruits and vegetables)	- Cook food until it is tender	- Sit quietly while eating
				- Sticky or tough foods (e.g., peanut/seed butters, meat, marshmallows, bubble gum)	- Cut food into small pieces	- Serve small portions
				- Firm, smooth or slippery foods (e.g., hot dogs, hard candies, berries, cherries)	- Remove bones, seeds and pits	- Encourage children to eat slowly and chew well
					- Cut round foods lengthwise	
U.S. Department of Agriculture (Infant Nutrition and Feeding. A Guide for Use in the WIC and CSF Programs) (24)	US	Revised in 2009	The guide was aimed at infants	Chunks of meat, hot dogs, fish with bones, chunks of cheese, nuts and seeds, nut/seed butters, beans, corn kernels, cherry and grape tomatoes, raw (or partially cooked) vegetables, grapes, berries, cherries, fruits with seeds or pits, pieces of canned fruit, popcorn, pretzels, chewing gum, sticky and hard candies		- Actively supervise children - Maintain a calm atmosphere - Encourage children to eat slowly
						- Avoid teething pain medicine before mealtimes
						- Do not give food in the car
Department of Health (19)	South Australia	2011	Under 4 years	- Food with skins (e.g., hot dogs)	- Remove bones, seeds, pips, stones, skins	- Make sure children are calm
				- Round-shaped foods (e.g., grapes)	- Cut into small pieces	- Eat sitting upright
				- Foods with stones, seeds or pips (e.g., cherries)	- Do not give corn chips, hard or sticky candies, popcorn, nuts and hard crackers	- Actively supervise children
				- Hard, crunchy or stringy food (e.g., crackers)		- Eat slowly and never force children to eat
				- Chewy and tough foods (e.g., meat)		
				- Foods with bones		
Health Canada, Canadian Pediatric Society, Dietitians of Canada, Breastfeeding Committee for Canada (17)	Canada	2014	From 6 to 24 months	Hard, small and round, smoothy and sticky, solid foods	- Grate raw carrots - Remove pits - Chop grapes - Thinly spread peanut butter - Chop foods with fibrous or stringy textures	- Actively supervise children - Do not feed children in the car
Scientific Advisory Committee on Nutrition (SACN) (18)	United Kingdom	2018	First year of life	Whole nuts		- Actively supervise children
WHO (25)		2005	From 6 to 24 months	Items with shape and/or consistency that make them get stuck in the trachea (e.g., whole nuts, whole grapes, raw carrots)	Gradually increase food consistency according to infant abilities	

These originated from the American Academy of Pediatrics (AAP) (15), the Canadian Pediatric Society (16, 17), the Scientific Advisory Committee on Nutrition of the UK (18), the governments of South Australia (19) and New Zealand (Ministry of Health) (20) and the United States Department of Agriculture (USDA) in the context of the Child and Adult Care Food Program (CACFP) (21–23), the Special Supplement Nutrition Program for Women Infants and Children (WIC) and the Commodity Supplemental Food Program (CSFP) (24), and the WHO (25).

Most of the recommendations are aimed at parents (families), childcare providers and nutritional counselors, while the policy statements of the AAP and the Canadian Pediatric Society provided recommendations not only for families and childcare providers but also for food manufacturers and authorities in charge of food safety [e.g., the Food and Drug Administration (FDA)].

### Hazardous Food Items: Characteristics

The guidelines agree that dangerous food products share specific shape, texture, and dimension characteristics. Both small (e.g., nuts and seeds) and large (e.g., pieces of raw fruits and vegetables) food items are dangerous: the former may reach the airway before the child can chew them, while the latter are difficult to move around in the mouth. Round-shaped (e.g., berries and whole grapes) and cylindrical (e.g., hot dogs) food products are the most dangerous shapes because they can easily become impacted in the upper airway (hypopharynx) if aspirated, obstructing it completely in the process. Among textures, the most dangerous ones are the hard, the sticky (e.g., peanut butter), the fibrous (e.g., celery), and the and compressible (e.g., hot dogs). Hard and fibrous textures are difficult to chew for small children due to their lack of teeth, particularly molars. Compressible food items are extremely dangerous because they can slip into the airway before the child chew them and conform to the hypopharynx, causing a complete obstruction. Sticky textures are difficult to remove if they get stuck in the airway.

All the guidelines provided detailed lists of hazardous foods, and some recommendations created different classes of dangerous food products that share similar textures, shapes, and dimensions.

In addition to solids, two of the guidelines promoted by the USDA (23, 24) also provided recommendations for preventing choking on liquids during bottle-feeding by avoiding propping up bottles in babies' mouths and using nipples with holes that are too large.

### Hazardous Food Items: Food Preparation

The guidelines agree that some of dangerous food items could be prepared in such a way that makes them safer, while others should be avoided until the child is developmentally mature enough to handle them (Table 2).

**Table 2:** Core recommendations in food preparation and serving derived from the overviewed guidelines.

**Dangerous food items (main categories)**	**Minimum age recommended**	**Recommended preparation**
Grapes, cherries, berries, cherry tomatoes, olives	-	Cut into small pieces. For grapes, cherries and olives, remove stones and pits.
Peanuts, nuts and seeds	4/5	Cut finely or grind.
Hot dogs and other cylindrical food items	-	Cut lengthwise and then into small pieces. Remove skins.
Peanut and other seed butters	-	Thinly spread onto bread.
Pieces of raw fruits and vegetables	-	Cook until tender or grate. Remove skins, fibers and stones.
Fish and meat	-	Cook until tender and then cut into small pieces. Remove bones and gristle.
Beans and peas	-	Cook until tender and then mash with a fork.
Candies, marshmallows, bubble gum	3/4	-

As a general rule, the guidelines agree in recommending (i) cooking food until it is soft (they also strongly emphasize the need for subsequent mashing with a fork); (ii) chopping round-shaped dangerous food items into small pieces; (iii) de-boning fish and meat; (iv) removing fibers from fibrous foods and then cutting them into small pieces; (v) never cutting cylindrical food products (e.g., hot dogs and carrots) into rounded pieces but instead cutting them lengthwise; (vi) spreading thick pastes thinly; and (vii) always removing seeds.

Sweets, marshmallows, and bubble gum are not recommended for children under the age of 3 because they pose a significant choking hazard and do not add any nutritional value to the child's diet. It may indeed be stated that such food items are not recommended as part of a healthy diet. Along the same lines, despite their great nutritional values, nuts and seeds as a whole are not recommended, as they have been demonstrated to be the most common cause of food choking injuries (26).

Despite small differences among the retrieved guidelines regarding the age at which it is possible to start offering dangerous food item (with age ranging from 3 to 5 years old), the basic message about which food items are the most dangerous and the methods of preparation needed to minimize choking hazards are generally agreed across all guidelines.

### Behavioral Rules at Mealtimes

In addition to food preparation, guidelines highlighted following simple behavioral rules during mealtimes to make them safer (Table 3).

**Table 3:** Behavioral rules at mealtimes.

**Behavioral rules**	**Behaviors to be avoided**
Make sure the child is eating at the table and seating upright	Do not feed children in the car or while they are playing or running
Make sure the child is relaxed	Do not give food to children while they are crying or laughing, avoid distractions
Actively supervise children while they are eating	Never leave children alone while they are eating
Eat slowly, give small servings, make sure the child chews well before swallowing	
Give food that is appropriate to child's developmental stage	Do not give difficult-to-chew food items to small children

The most important is to supervise children actively and not leave them alone while eating. However, because not all choking episodes can be prevented, guidelines recommend that adult supervisors (both family members and childcare providers) learn to perform pediatric cardio-pulmonary resuscitation (CPR) as well as the appropriate pediatric maneuvers for cases where a child is choking.

### Food Choking Prevention: Beyond Food Preparation

As is noted in the AAP's policy statement (15), most hazardous food items are manufactured by man (e.g., hot dogs, gel candies, and bubble gum). This highlights the fact that, in addition to recommendations for families and childcare providers on dangerous food items and their preparation, it is essential to inform public authorities of the need to develop and implementing regulations aimed at food manufacturers. The primary prevention strategies, as have been recommended by the AAP (15) and the Canadian Pediatric Society (16), should include different interventions, including (i) mandatory warning labels on dangerous food products, (ii) permanent surveillance systems allowing for the prompt recognition of hazardous foods (e.g., new products in the market), (iii) recalls of dangerous food products from the market if the choking risk they pose is unacceptable, and (iv) designing/redesigning food products to minimize choking hazards. Other useful activities may include compulsory training on the primary and secondary prevention of choking injuries for childcare providers and foodservice employees (e.g., restaurants and cafeterias), as was done in the SafeFood4Children (SF4C) program (27).

### Nutritional Implications of Recommendations for Food Choking Prevention

Among hazardous food items, we may identify food manufactured by man (e.g., hot dogs, gel candies, and bubble gum) and raw ones (e.g., some types of fruits and vegetables, nuts and seeds). Undisputedly, raw food recognized to be at risk of choking is nutrient dense, contributing to a healthy nutrient intake in young children. A misinterpretation of recommendations for food choking prevention may lead to an inappropriate nutrient intake, which may have detrimental effects on the growth of the child. However, apart from rare exceptions (e.g., nuts and seeds, which pose an unacceptable choking hazard), raw dangerous food items may be given to children by modifying those characteristics associated with a high risk of choking. Food preparation must be adequate to the age of the child, who should taste different textures with the increased developmental skills, as it has been recommended by the WHO recommendations on the introduction of solids (25, 28).

## Discussion

The present paper aims to provide an overview of the international recommendations for preventing food choking injuries because young children are at high risk of choking on food due to their psycho-physiological characteristics (29). Despite not having any expectation of being exhaustive and the intrinsic difficulty of setting a clear line between governmental and non-governmental guidelines and—for some countries—accessing documents in the native language, our overview still collected and analyzed perhaps the most detailed set of recommendations in the field.

From a public health perspective, our overview stressed the importance of developing primary prevention policies to regulate dangerous food products in the market and to sensitize adult supervisors. Such health planning interventions have proven to be highly effective in preventing injuries in the pediatric age group. For toy safety, the implementation of strict legislation and quality standards, in combination with the development of public campaigns to sensitize the population about the importance of choosing toys and nursery products whose safety has been certified by international standards, had resulted in a dramatic decrease in the burden of injuries due to toys in recent decades (30).

Notwithstanding, no consistent and coordinated interventions have yet been undertaken about food choking injuries. Certainly, the topic of food choking is much more diversified and complex than that of a simple injury since it involves a variety of products and their preparation, both for industrial and domestic use.

In this case, the intersectional understanding of a certain reality, a healthcare-related issue, results in the most appropriate paradigmatic and pragmatic choice either on a theoretical or methodological level. Most recent research underlined how specific educational policies promote the best strategies to cope with a healthcare problem, including that of choking injuries (31).

Consequently, such an intersectional perspective should also be applied to understanding the causes underlying choking injuries in the pediatric age.

In Italy, about 600 severe cases leading to hospitalization are observed each year (1). Thus, in 2016, the Italian Ministry of Health, Directorate-General for Food Hygiene and Safety and Nutrition, established a technical working group with the explicit mandate to develop a set of recommendations that could drive consumers and public and private bodies to reduce the risk and its consequent burden of such injuries in children.

The recommendations, agreed as a consensus within the working group, and then evulgated in 2017 (32) were organized in two parts: (i) the recommendations on the food dimensions and preparation recommended to children of various ages (based on the present work) and (ii) a set of indications on best-practices targeted to industry, public bodies and the general public. The second part is innovative in introducing some novel concepts concerning the existing guidelines. For industry and the food-chain suppliers, there's the recommendation to add, on a voluntary basis, well-visible warnings on the risk of choking, in line with the recommendation of the AAP (15) but also, to adopt all technological approaches available to limit, starting from the “food design” phase, the products characteristics which may be associated with an increased risk of choking. The Italian Ministry Guidelines introduced a “reversal order of proof” to industry and food-chain suppliers: in case of an injury event, they should be—in principle—in charge of proving that everything reasonable was done to make the event extremely unlikely to happen. The other relevant innovation regards the introduction of the notion of epidemiological surveillance for those injuries, recommending to both families and bodies, reporting the observed injuries in a dedicated registry.

## Conclusions

It is essential that nutrition meets injury prevention and education by combining general recommendations about children's nutrition with those related to choking prevention to ensure both quality and safe diet. With the development of such guidelines, it is crucial to properly communicate this information to the population (risk communication), highlighting the importance of striking the right balance between safe and nutrient-dense food. Demonizing food items at high risk of choking (e.g., referring to them as “killers”) may contribute to the misunderstanding of these recommendations and promote wrong nutritional approaches for children. Stakeholders must be made aware of hazardous food categories and that these foods may be safely given to young children (apart from rare exceptions) by following recommendations for food preparation. The problem of food choking injuries represents a relevant public health issue that cannot be ignored, and national governments should adequately address that. We will only be able to reduce the burden of food choking injuries in the pediatric age group by strictly regulating this phenomenon and deeply sensitizing the population to it.

## Author Contributions

DG: conceptualization. GL, GBV, and SB: investigation. GBV: visualization. GL: writing—original draft. AH, RV, AF, GC, HR, AG, and SA: writing—review and editing. All authors contributed to the article and approved the submitted version.

## Funding

This work was partially supported by an unrestricted Grant from the Italian Ministry of Foreign Affairs, the Directorate General for Country Promotion, the Italian Ministry of Health, the Directorate General for Food Hygiene and Safety and Nutrition, and Prochild Onlus (Italy).

## Conflict of Interest

The authors declare that the research was conducted in the absence of any commercial or financial relationships that could be construed as a potential conflict of interest.

## Publisher's Note

All claims expressed in this article are solely those of the authors and do not necessarily represent those of their affiliated organizations, or those of the publisher, the editors and the reviewers. Any product that may be evaluated in this article, or claim that may be made by its manufacturer, is not guaranteed or endorsed by the publisher.
